# Re-Punching Tissue Microarrays Is Possible: Why Can This Be Useful and How to Do It

**DOI:** 10.3390/microarrays4020245

**Published:** 2015-05-11

**Authors:** Aurélien Lacombe, Vincenza Carafa, Sandra Schneider, Melanie Sticker-Jantscheff, Luigi Tornillo, Serenella Eppenberger-Castori

**Affiliations:** Molecular Pathology Division, Institute of Pathology, University Hospital of Basel, CH-4031 Basel, Switzerland; E-Mails: aurelien.lacombe@usb.ch (A.L.); vincenza.carafa@usb.ch (V.C.); sandra.schneider@usb.ch (S.S.); melanie.sticker@usb.ch (M.S.-J.); luigi.tornillo@unibas.ch (L.T.)

**Keywords:** TMA, H&E, IHC, ROI, TMA-M, TMA-GM, FFPE

## Abstract

Tissue microarray (TMA) methodology allows the concomitant analysis of hundreds of tissue specimens arrayed in the same manner on a recipient block. Subsequently, all samples can be processed under identical conditions, such as antigen retrieval procedure, reagent concentrations, incubation times with antibodies/probes, and escaping the inter-assays variability. Therefore, the use of TMA has revolutionized histopathology translational research projects and has become a tool very often used for putative biomarker investigations. TMAs are particularly relevant for large scale analysis of a defined disease entity. In the course of these exploratory studies, rare subpopulations can be discovered or identified. This can refer to subsets of patients with more particular phenotypic or genotypic disease with low incidence or to patients receiving a particular treatment. Such rare cohorts should be collected for more specific investigations at a later time, when, possibly, more samples of a rare identity will be available as well as more knowledge derived from concomitant, e.g., genetic, investigations will have been acquired. In this article we analyze for the first time the limits and opportunities to construct new TMA blocks using tissues from older available arrays and supplementary donor blocks. In summary, we describe the reasons and technical details for the construction of rare disease entities arrays.

## 1. Introduction: TMA Technology and Statistical Considerations

Tissue microarray (TMA) technology was first described by Battifora *et al.* [1] in 1986 as a ‘sausage’ block method to prepare multi tissue sections; it was used and modified by Wan *et al.* [2] in 1987, introducing the cannula, and further developed as a device that could rapidly produce TMA with regular shape and defined tissue size by Kononen [3] *et al.* in 1998. In the last fifteen years, technological progress and extensive biological and medical knowledge improvements are inducing researchers to continuously investigate putative diagnostic, prognostic and therapeutic biomarkers [4]. Traditional histopathology techniques are often unacceptably time consuming, extremely labor intensive and very expensive [5], while TMAs are a powerful tool to overcome many of these challenges and are often used for characterization of antibodies, tissue and disease specific epidemiological expression profiling of proteins via immunohistochemistry (IHC) and genes via *in situ* hybridization, respectively. Therefore, TMAs are more and more becoming the method of choice for large scale and epidemiological biomarkers investigations. IHC analyses of such TMAs have the great advantage to minimalize all the intra-laboratory differences in staining procedures, as well as the inter‑observer evaluation variability. Moreover, TMAs can even be constructed from paraffin needle biopsy specimens and also allow testing the expression of biomarkers on the following slides of a constructed TMA block for studying markers co-expressions [6,7,8,9,10,11,12,13]. 

Subsequently, several instruments were developed reaching more and more technical improvements. These include the TMA Master^®^ (TMA-M; 3D-Histech Ltd, Sysmex Suisse AG, Horgen, Switzerland) and more recently a unique today’s available system: the TMA GrandMaster^®^ (TMA-GM; 3D-Histech Ltd, Sysmex Suisse AG). Today, the TMA-GM is the most automated and sophisticated device for the construction of TMAs, allowing the precise selection of specific regions of interest (ROI) on the donor blocks. It detects the depth of the donor blocks and drills each time the most appropriate hole in the empty paraffin recipient blocks. It acquires a tissue carrot from the donor blocks and places it at the assigned coordinate (X-Y) and it allows the construction of up to 12 recipient blocks in parallel. Thanks to the high precision of this instrument, we hypothesized and demonstrated that previously prepared TMAs (independently from their mode of construction) could be used as donor blocks in order to transfer special tissue cores, when necessary.

When planning a new study, histological, statistical and logistical aspects must be considered [14,15,16,17]. These comprehend tissue type, specific histological regions and cell types for inclusion in the TMA, the number of tissue spots, sample size, and statistical analysis [18,19]. Quality of the samples and sample size represent the most challenging issue encountered and are often limiting the investigation. Moreover, it happens that information at time of planning is insufficient and/or that during the performance of such a study—thanks to knowledge resulting in the course of the study itself or concomitantly discovered by others—subpopulations of particular scientific and medical interest are identified [20]. Thanks to the general improvement of all technologies, leading to brighter biomedical knowledge, these rare cohorts of patients become increasingly topic of new research interest, though in general still not predictable at time of TMA design [21,22,23,24]. Statistical power of analysis based on, for example, future therapy response rate of a certain patient cohort could probably reveal the need to select tissues of certain patients from existing, ongoing studies and group them into a new one for obtaining significant results. In that case processing of such tissue cores could be essential for the successful performance of a new clinically relevant study and otherwise wasted if not specifically used on the original TMA. With this purpose we planned the following experiments for tissue core “*transplantation*” and defined optimal conditions for the construction of new “*elastic*” TMAs. To our knowledge no other work along these lines has previously been performed.

## 2. Experimental Section: Study Design

**Figure 1:**
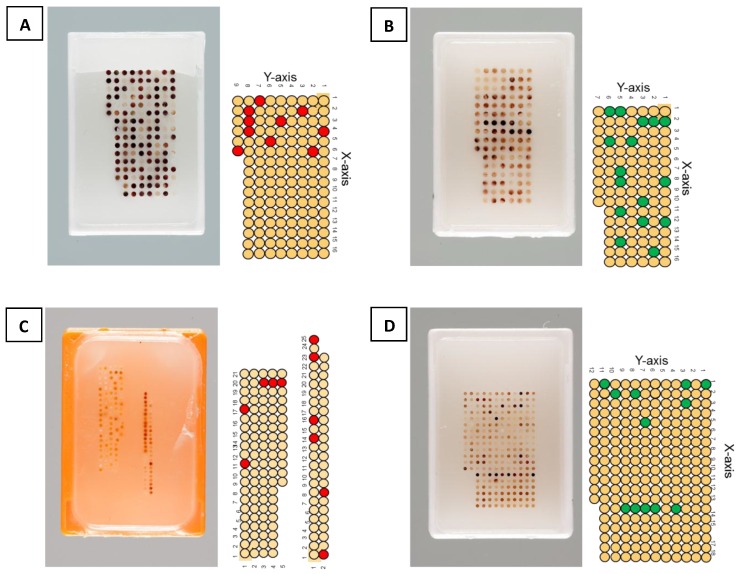
Schematic overview of TMA donor blocks. (**A**) TMA performed by TMA-M (core diameter is 1 mm, and distance between cores is 0.5 mm). (**B**) TMA performed by TMA-GM (core diameter is 1 mm, and distance between cores is 0.7 mm). (**C**) TMA performed by old machine (core diameter is 0.6 mm, and distance between cores is 0.6 mm). (**D**) TMA performed with TMA-GM (core diameter is 0.6 mm, and distance between cores is 0.6 mm). Red and Green circles represent the selected tissues in each TMA donor block to be re-punched and transferred to a new recipient block. (For donor TMAs with 1mm diameter tissues, red circles represent tissues punched with TMA-M (A), and green circles represent tissues punched with TMA-GM (B). For donor TMAs with 0.6 mm diameter tissues, red circles represent tissues punched with original TMA instrument (C), and green circles represent tissues punched with TMA-GM (D).

For the purpose of this review we created several TMAs under defined conditions. Two of them (Figure 1B,D) were built by means of the TMA-GM instrument. Moreover, we took an old tissue array (Figure 1C) prepared ten years ago with the original TMA machine developed in 1998 by Beecher *et al.* and modified by Mirlacher [25] and another array (Figure 1A) constructed in 2013 with a TMA-M device. In addition, we selected donor blocks representative for various organs and histopathological characteristics. 

**Figure 2:**
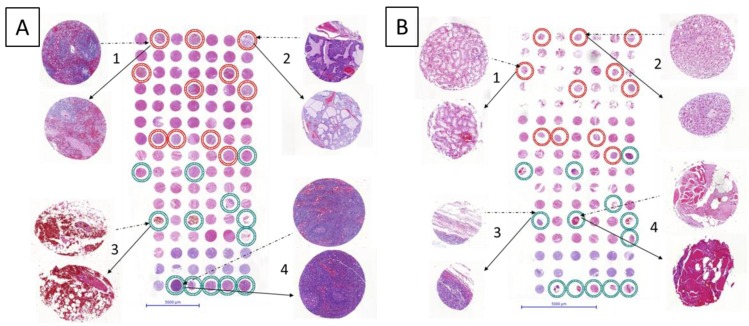
Result of re-punched TMA with 1mm core diameter (**A**) and 0.6 mm core diameter (**B**), respectively. Distance between cores is 0.6 mm in both cases. The circles indicate tissue provenance. In the TMA with 1mm core diameter (A), red circles represent tissues originally punched with TMA-M (Figure 1A), and green circles represent tissues originally punched with TMA-GM (Figure 1B). In TMA with 0.6 mm diameter (B), red circles represent tissues originally punched with original TMA instrument (Figure 1C), and green circles represent tissues originally punched with TMA-GM (Figure 1D).

Array layout was defined indicating the number and location of tissue core to be collected from each donor block, their positions on the array to be constructed (recipient block), the diameter of each carrot and the distance between each spot on the recipient block. Several tissues were selected from the donor TMAs to be re-punched and transferred to the new TMA. Green and red circles indicate the selected tissues to be transferred (Figure 1) and their positions on the newly created TMAs (Figure 2). 

Once the new TMA was built, the block was extracted from the TMA-GM and put on a glass slide. With the thumb and trigger finger the TMA block was pressed to the glass slide. The block up-side-down on the glass slide was incubated overnight at 42 °C. In the morning, after the TMA block was left to cool at room temperature (RT) for approximately 30 minutes and the glass slide removed, and the block was ready to be used. 

A section of each donor block was cut using a standard microtome. The slides were stained with Haematoxylin and Eosin (H&E) and scanned. The pictures were checked by the pathologist on the computer screen by means of the Panoramic Viewer^®^ (3D-Histech, Sysmex AG) program. The designed layout was loaded on TMA-GM. Images of donor blocks and corresponding histological slides were overlaid using the TMA-GM software. The designated tissue spots on the donor TMAs were directly selected according to the respective array coordinates (Figure 1 and Figure 2). Further slides of the donor blocks, as well as of the newly created TMA blocks with transferred tissues were stained with five antibodies: CD34 (Ventana 790-2927), CD45 (Ventana 760-4279), Smooth Muscle Actin (1A4; Ventana 760-2833), pan-Keratin (Ventana 760-2595) and Ki67 (Dako IR626). All stainings were performed with the BenchMark XT (Ventana Medical System Inc, Tucson, AZ, USA). 

## 3. Results and Discussion

In the present work, we constructed two new TMAs taking the tissue punches from normal FFPE donor blocks as well as from previous arrays constructed with different instruments. The experiment was carried out with punches of 1 mm and 0.6 mm diameter, respectively, these two sizes being the most commonly used in ongoing translational research TMA approaches. 

**Figure 3:**
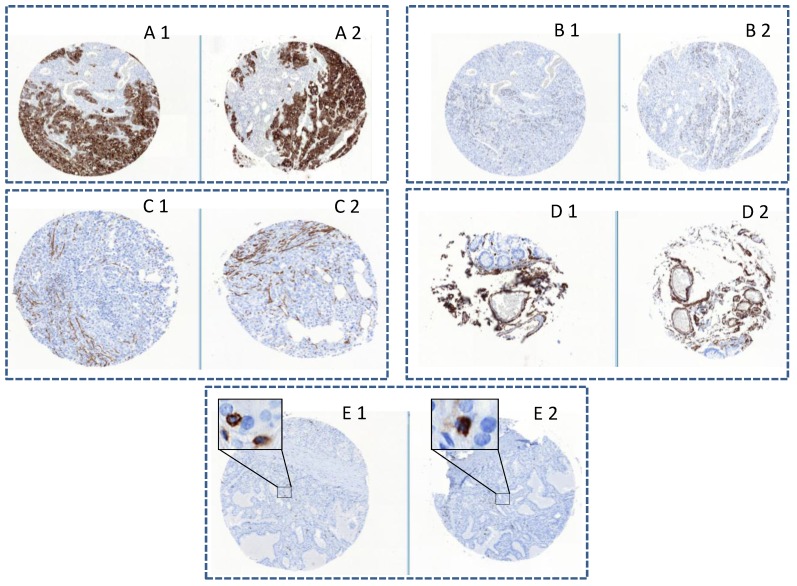
Comparison of protein expressions in the same tissue cores before (**1**) and after re-punching (**2**): (**A**) Pan-keratin in epithelial carcinoma cells (1mm diameter core); (**B**) Ki67 in epithelial carcinoma cells (1 mm diameter core); (**C**) Actin positivity of smooth muscle (0.6 mm diameter core); (**D**) Colon with blood vessels positive for CD34 (0.6 mm diameter core); and (**E**) Thyroid carcinoma with CD45 positive lymphocytes (1 mm diameter core).

As shown in Figure 2 the resulting new TMAs were successfully built. The tissue spots on H&E slides of the new TMAs were compared to the ones of the original TMAs. The re-punched tissues retained histological characteristics independently from the organs. Even hemorrhagic adipose tissue was successfully transferred (Figure 2A-3). 

Some cores appear slightly compressed (Figure 2B-2,3); the H&E staining revealed that some spots were less round compared with the one of the original TMA. This rare observation occurred exclusively in tissues with 0.6 mm diameter independently from the machine used for the original TMA construction and the age of the TMA donor blocks. Notably, these small variations do not affect the intrinsic histopathological tissue characteristics and can be in part due to the cutting of the TMA slide. Thus the usefulness of the “transplanted” tissue cores is guaranteed. 

Example cores for both tissue arrays are shown in detail. Dotted line arrows point from images of the donor block to the core on the recipient TMA. Full line arrows connect the re-punched core to its magnified image. In the 1 mm TMA: A-1. spleen; A-2. pancreas; A-3. hemorrhagic adipose tissue; A‑4. lymph node. In the 0.6 mm TMA: B-1. renal medulla; B-2. liver; B-3. epithelial tumor; and B-4. striated skeletal muscle. 

Figure 3 illustrates the successful IHC staining obtained using five different tissue markers. The examples reported are completely representative for all transferred cores. The obtained similarity in staining of all type of tissues independently from the size of the cores, the original machine used, and the age of the block is impressive. These results underline the precision of the tissue transfer and the fact that the epitopes remain unchanged after a second overnight incubation at 42 °C. 

**Figure 4:**
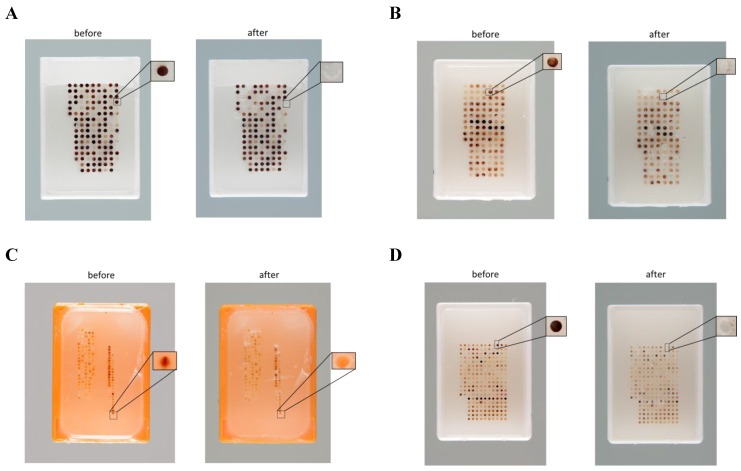
Donor array, before and after tissue transfer, showing unimpaired remaining cores. (**A**) TMA performed with TMA-M (core diameter is 1 mm, and distance between cores is 0.5 mm); (**B**) TMA performed with TMA-GM (core diameter is 1 mm, and distance between cores is 0.7 mm); (**C**) TMA performed with old machine (core diameter is 0.6 mm, and distance between cores is 0.6 mm); and, (**D**) TMA performed with TMA‑GM (core diameter is 0.6 mm, and distance between cores is 0.6 mm).

The tissues were in general completely transferred. The TMA-GM can precisely center a tissue carrot even in case of 0.6 mm cores. In Figure 4 the spots on the TMAs of origins are shown before and after the tissue transfer. The holes after tissue transfer are completely empty in all cases. 

The only limiting factor of this re-punching process is the distance between the tissue cores in the donor TMA. We tested TMA with 0.6, 1 and 2 mm cores’ diameters and distances between cores ranging of 0.5, 0.6 and 0.7 mm. The TMA-GM is very precise and can export any kind of tissue carrots even when these lay very close to each other. However, if the gap between the cores is less than 0.6 mm and many cores in a row have to be re-punched, the bridges between the empty cores lose stability. Therefore, we recommend designing new arrays with an intra-core distance of 0.6 mm. However, since many existing TMAs are constructed with distance less than 0.6 mm, we took inspiration from previously described practical methodologies [26,27] and tested manually refilling of empty holes with paraffin by means of an appropriate syringe. In order to fulfill the aim of stabilizing the donor TMA, the manual refilling of holes in a row or otherwise adjacent to each other must occur after the export of each single tissue. That way, the paraffin helps stabilizing partially the thin bridge that otherwise would result between two empty holes. Subsequently, the next tissue specimen will remain vertical allowing a precise transfer. Nevertheless, this manual refilling requires time, depends on personal ability and, the inserted paraffin will not guarantee the complete stable filling of the empty hole. In this case the usefulness of re-punching and the risk of destabilizing an existing TMA should be carefully evaluated. 

## 4. Conclusions

TMA technnology has revolutionized translational research allowing the simultaneous, standardized and reproducible analysis of many tissues from any entities. TMAs are intended for the processing of hundreds of tissue specimens and adequate for large epidemiological studies. During the performance of such studies rare but very interesting and important subpopulations are often identified, thus prompting scientists to separate investigation of such rare cohorts. 

The development of new extremely precise instruments, like the TMA-GM machine, allows to take material from donor blocks with extreme precision. In the present article, we describe the successful transfer of tissue cores from existing TMAs to new ones, relocating tissue of interest together with others obtained from “normal” FFPE donor blocks. This approach is useful and can be successfully practicable using any type of “donor” TMAs, if the distance between the cores does not destabilize the “donor” TMA structure in case that several tissues to be transferred are adjacent to each others. 
